# Developments and challenges of FLT3 inhibitors in acute myeloid leukemia

**DOI:** 10.3389/fonc.2022.996438

**Published:** 2022-09-14

**Authors:** Shuai-Shuai Ge, Song-Bai Liu, Sheng-Li Xue

**Affiliations:** ^1^ National Clinical Research Center for Hematologic Diseases, Jiangsu Institute of Hematology, The First Affiliated Hospital of Soochow University, Suzhou, China; ^2^ Institute of Blood and Marrow Transplantation, Collaborative Innovation Center of Hematology, Soochow University, Suzhou, China; ^3^ Suzhou Key Laboratory of Medical Biotechnology, Suzhou Vocational Health College, Suzhou, China

**Keywords:** AML, FLT3 inhibitors, targeted therapy, drug resistance, mechanisms of resistance

## Abstract

FLT3 mutations are one of the most common genetic alterations in acute myeloid leukemia (AML) and are identified in approximately one-third of newly diagnosed patients. Aberrant FLT3 receptor signaling has important implications for the biology and clinical management of AML. In recent years, targeting FLT3 has been a part of every course of treatment in FLT3-ITD/TKD-mutated AML and contributes to substantially prolonged survival. At the same time, wide application of next-generation sequencing (NGS) technology has revealed a series of non-canonical FLT3 mutations, including point mutations and small insertions/deletions. Some of these mutations may be able to influence downstream phosphorylation and sensitivity to FLT3 inhibitors, while the correlation with clinical outcomes remains unclear. Exploration of FLT3-targeted therapy has made substantial progress, but resistance to FLT3 inhibitors has become a pressing issue. The mechanisms underlying FLT3 inhibitor tolerance can be roughly divided into primary resistance and secondary resistance. Primary resistance is related to abnormalities in signaling factors, such as FL, CXCL12, and FGF2, and secondary resistance mainly involves on-target mutations and off-target aberrations. To overcome this problem, novel agents such as FF-10101 have shown promising potential. Multitarget strategies directed at FLT3 and anomalous signaling factors simultaneously are in active clinical development and show promising results.

## 1 Introduction

Acute myeloid leukemia (AML) is a clonal stem cell malignancy that is characterized by infiltration of abnormally differentiated myeloid progenitor cells (blasts) and has a historically high mortality rate (1, 2). FLT3 (FMS-like tyrosine kinase-3) is a type 3 receptor tyrosine kinase (RTK) that consists of five Ig-like domains in the extracellular region, a juxtamembrane (JM) domain, and an interrupted kinase domain in the intracellular region (3). In normal cells, FLT3 is mainly expressed in hematopoietic stem or progenitor cells and plays an important role in hematopoietic expansion by binding the FLT3 ligand (FL) (4). Most primary AML cells also express FLT3, accompanied by FL stimulation leading to proliferation and anti-apoptosis of AML cells (5–7).

FLT3 is one of the most commonly mutated genes in acute myeloid leukemia, accounting for 15%-35% of newly diagnosed patients (8). Most of these mutations are internal tandem duplication (ITD) mutations that insert into the juxtamembrane region and tyrosine kinase domain 1, while activating point mutations localized in the tyrosine kinase domain activating loop (TKD) are less frequently observed. ITD and TKD mutations occur in approximately 20% and 7% of AML patients, respectively (9, 10). FLT3 mutations in AML are of clinical significance. FLT3-ITD mutations are strongly linked to worse clinical features and poor prognosis, making FLT3-ITD an independent prognostic marker. while the presence of FLT3-TKD mutations is correlated with a favorable prognosis over FLT3-ITD-mutated patients (10–13). With the wide application of NGS, an increasing number of non-canonical activating point mutations and indel alterations have been detected, but it is unclear whether those non-canonical FLT3 alterations are associated with prognosis because of their low incidence (14–18).

In the era of targeted therapy, mutant FLT3 serves as a promising molecular target spot for the treatment of AML, and great changes have been made in the clinical management of FLT3-mutated AML due to the development of FLT3 inhibitors (13, 19, 20). However, drug resistance remains a challenge despite FLT3 inhibitors providing a dramatic therapeutic response in the frontline and relapsed/refractory settings (21–24). This review mainly focuses on the recent progress in applying FLT3 inhibitors in AML and the mechanisms of drug resistance.

## 2 Classification of FLT3 inhibitors

FLT3 inhibitors can be classified using two primary schemas: generation and type. The first-generation FLT3 inhibitors were tyrosine kinase inhibitors (TKIs) with multi-kinase target activity. Existing first-generation TKIs include lestaurtinib (CEP-701), sunitinib (SU11248), midostaurin (PKC412), and sorafenib (BAY43-9006) (25–28). The antileukemic effects of these multi-kinase inhibitors likely derive from the simultaneous inhibition of FLT3 and parallel pathways, but multiple off-target effects also bring about increased toxicities (29). Subsequently, second-generation FLT3 inhibitors with higher selectivity and inhibitory activity were identified. Second-generation FLT3 inhibitors can more selectively inhibit FLT3, and thus have greater clinical potential and fewer off-target effects. Second-generation FLT3 inhibitors include gilteritinib (ASP2215), quizartinib (AC220), and crenolanib (CP868596). In plasma, first-generation inhibitors have higher IC50 values and shorter half-lives than their second-generation counterparts, which explains their limited clinical potency (30–32).

Furthermore, these FLT3 inhibitors can be roughly classified into two types according to the binding mode to FLT3. Type I inhibitors bind to the ATP-binding site in the intracellular active pocket and enable downregulation of the phosphorylation of both ITD and TKD mutations. In contrast, because type II inhibitors are designed to favorably bind to the hydrophobic space of the inactive conformation of FLT3, they are made inaccessible by TKD mutations (24). The details of established FLT3 inhibitors are summarized in **Table 1**.

**Table 1:** Established FLT3 inhibitors and features.

Inhibitor name	Generation	Type	IC50 in plasma(nM)	Half life *in vivo* (h)	Clinical development	Observed mechanisms of secondary resistance	
						On-target mutations	downstream/parallel signal pathways abnormalities
Midostaurin	First	I	1000 (28)	5-29 (33)	Approved for newly diagnosed AML by FDA in 2017	FLT3-N676K/D/S, F691I/L, G697R/S mutations (34, 35)	Upregulation of MCL-1 (36), AXL (37)
Sorafenib	First	II	308 (38)	20-38 (39)	Phase III	FLT3-D835Y, F691L, Y842H, A848P mutations (40, 41)	Upregulation of PIM (42), ERK (43)
Gilteritinib	Second	I	17-33 (31)	45-159 (44)	Approved for R/R AML by FDA in 2018	FLT3-F691L, Y693C/N, G697S, N701K mutations (45–47)	Mutations in N-Ras signaling (45, 48)
Quizartinib	Second	II	18 (49)	36+ (50)	Approved for R/R AML by MHLW in 2019	FLT3-D835Y, F691L, Y842D mutations (40, 41)	Upregulation of PIM (42), ERK (43), AXL (37, 51)
Crenolanib	Second	I	48 (32)	8 (32)	Phase III	FLT3-K429E, F691L, N701K mutations (46, 52)	N-Ras, IDH2, TET2 mutations (48, 52)

## 3 FLT3 inhibitors for AML therapy

### 3.1 Therapy for canonical FLT3 mutated AML

**Figure 1:**
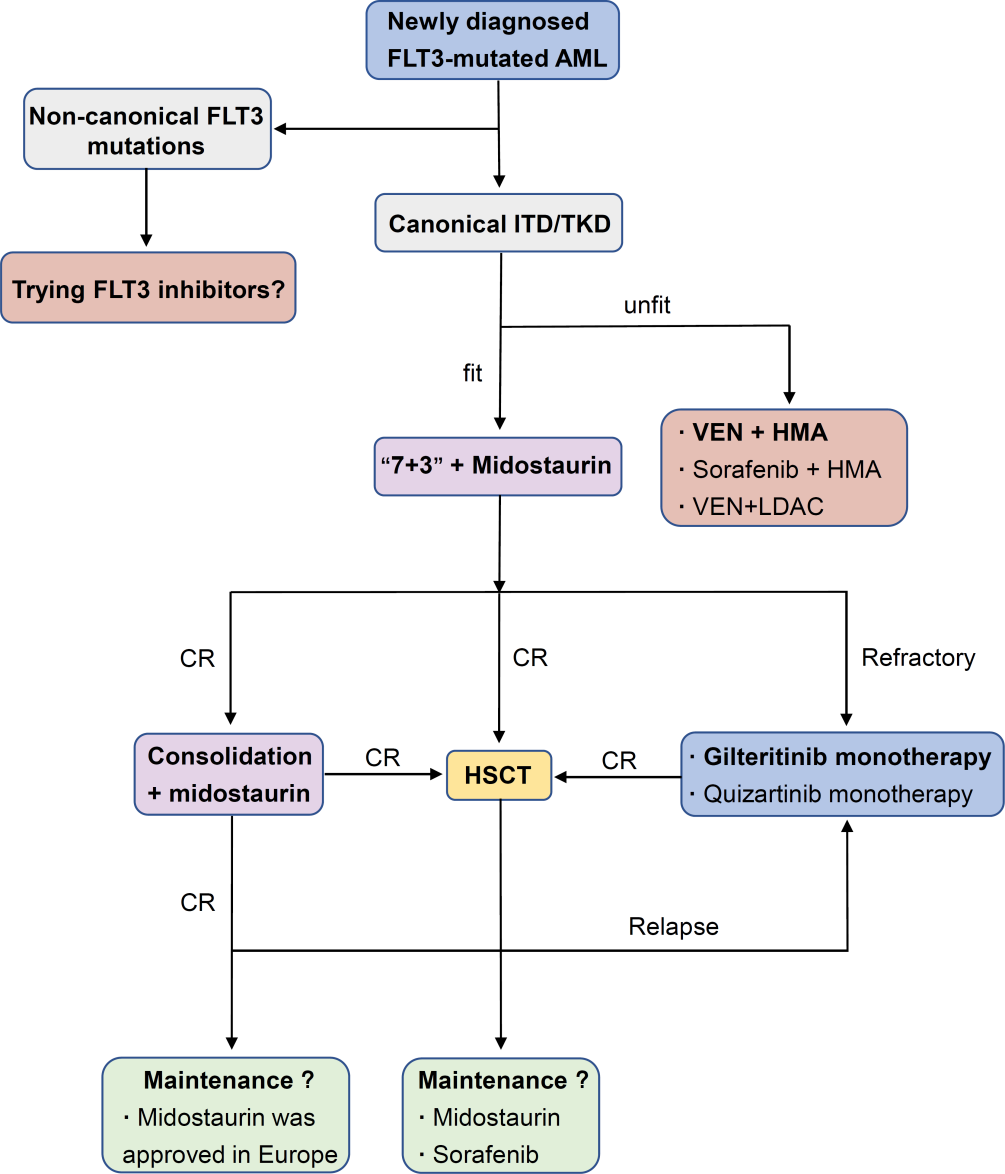
Applications of FLT3 inhibitors in AML.

Historically, except for acute promyelocytic leukemia (APL), conventional chemotherapy (“3+7”) alone was the standard strategy for FLT3-mutated AML. Although FLT3-ITD AML had a similar response to induction chemotherapy compared to WT counterparts, a shorter duration of remission and higher relapse rate attracted attention (53, 54). Preliminary results from large multicenter trials showing a survival improvement from a combination of chemotherapy and FLT3 inhibition (compared with historic controls) made this approach look promising (25, 55). After continuous optimization of treatment strategies and renewal of FLT3-targeted drugs, FLT3 inhibitors have already been widely used in the clinical treatment of FLT3-mutated AML (**Figure 1**).

#### 3.1.1 Induction and consolidation therapy

The fitness and age of patients are key points in the decision for the management of newly diagnosed AML with FLT3 mutations. Midostaurin plus standard chemo-therapy is the first choice for those who are fit. In the phase III RATIFY trial, young adult patients (16-59 years old) with FLT3-ITD and FLT3-TKD were randomly assigned to receive standard induction and consolidation chemotherapy plus either midostaurin or placebo. Subsequently, those who reached remission after consolidation therapy received either midostaurin or placebo as maintenance. This combination resulted in significant improvement in event-free survival (EFS; 8.2 vs. 3.0 months, P=0.002) and median overall survival (OS; 74.7 vs. 25.6 months, P = 0.009) compared to chemotherapy alone, although a larger population with an FLT3-TKD mutation in the RATIFY study than that seen in the general population might bias the clinical outcomes toward this less aggressive subtype. Regrettably, the complete remission (CR) rate was not obviously improved (21). In 2017, midostaurin, one of the first FLT3 inhibitors to be studied in AML, was approved for use with induction and consolidation by the US Food and Drug Administration (FDA), as administered in the RATIFY trial, and com-bination therapy was recommended for the preferred strategy by the guidelines of the National Comprehensive Cancer Network (NCCN).

Older adults (>60 years old) who cannot undergo intensive chemotherapy often receive less intensive regimens, including hypomethylating agents (azacitidine or decitabine), because of limited effective treatment options. Hypomethylating agents and low-dose cytarabine were associated with poor CR plus CRi rates and median survival times (56, 57). Venetoclax, a selective small-molecule Bcl-2 inhibitor, has demonstrated an outstanding ability to induce apoptosis of AML cells *in vitro* (58, 59). In the phase III VIALE-A trial, treatment with azacitidine and venetoclax obviously improved the remission rate of various mutated subgroups and the median OS (60). However, the clonal evolution of FLT3-ITD loss of heterozygosity (LOH) may lead to treatment failure (12). As an alternative strategy, low-intensity chemotherapy (azacitidine or decitabine) plus sorafenib and venetoclax in combination with LDAC showed poor performance (61, 62).

However, it is important to note that the combination of venetoclax and FLT3 inhibitors shows great potential. The combination of FLT3 inhibitors (gilteritinib or sorafenib) with venetoclax could synergistically reduce cell proliferation and enhance apoptosis/cell death in FLT3/ITD cell lines and primary AML samples. Venetoclax was also able to attenuate FLT3 inhibitor-resistance of cells to gilteritinib or sorafenib treatment by inhibiting the MAPK/ERK pathway (63). In a phase II trial, 25 older patients with FLT3 mutated AML were enrolled and accepted triplet therapy combining FLT3 inhibitor, venetoclax, and HMA. The composite complete remission (CRc) rates in patients with newly diagnosed AML achieve 92% and 62% in R/R patients (64). Moreover, a retrospective analysis of their team demonstrated that older and unfit adult patients with newly diagnosed FLT3 mutated AML receiving a triplet regimen (lower intensity chemotherapy + FLT3 inhibitor + venetoclax) had a significantly higher CR/CRi rate (93% vs. 70%, P = 0.02) and longer median overall survival (NR vs. 9.5 months, P < 0.01) compared with doublet (lower intensity chemotherapy + FLT3 inhibitor) regimens (65). Overall, the combination of venetoclax and FLT3 inhibitors may be an effective frontline regimen for FLT3 mutated AML, which should be further validated prospectively.

#### 3.1.2 Maintenance therapy

The role of FLT3 inhibitors in maintenance therapy is intriguing, either during re-mission for patients who do not accept HSCT or for those who are undergoing HSCT. Data from several clinical trials that included TKI maintenance therapy after first-line induction and consolidation suggest that it may be a promising approach (21, 66–69).

**• Post-chemotherapy maintenance therapy**

In the notable phase III RATIFY study, patients accepted up to one year of midostaurin maintenance following induction and consolidation chemotherapy plus midostaurin, while FLT3 inhibitor maintenance was discontinued once patients underwent HSCT. An unplanned *post hoc* efficacy analysis of the midostaurin maintenance phase in the RATIFY trial suggested that midostaurin maintenance might not further reduce the probability of relapse, although it was well tolerated (21). Maintenance of midostaurin after chemotherapy did not receive US FDA approval due to the result of limited efficacy shown from clinical data. In the SORAML study, maintenance therapy with sorafenib after chemotherapy ameliorated RFS, though the trial could not determine to what extent sorafenib maintenance influenced RFS (66). Recently, a long-term follow-up study validated a clear benefit in RFS; however, it did not translate into an OS benefit (67). To evaluate actual benefits from maintenance therapy of FLT3 inhibitors, separate randomized trials will compare gilteritinib or quizartinib vs. placebo maintenance for up to two and three years following chemo-therapy, respectively (NCT02927262, NCT02668653).

**• Post-HSCT maintenance therapy**

Allogeneic hematopoietic stem cell transplantation (allo-HSCT) is considered the most powerful method for hematopoietic malignancies. Allo-HCST improves the out-come of AML patients with FLT3-ITD AML, but leukemia relapse remains a frequent factor of failure (70–72). In the post-HSCT maintenance setting, recent evidence has shown the antileukemic synergism of FLT3 inhibitors. In a phase I hypothesis-generating trial, midostaurin was added to intensive chemotherapy followed by allogeneic hematopoietic cell transplantation (allo-HSCT) and midostaurin maintenance therapy for 12 months. This study demonstrated a significant improvement in EFS by midostaurin compared to 415 historical controls (73). Sorafenib maintenance after HSCT in a randomized, placebo-controlled, double-blind phase II trial also showed po-tential improvement in RFS (24-month RFS, sorafenib vs. placebo, 85.0% vs. 53.3%, P=0.002) and OS (24-month OS, sorafenib vs. placebo, 90.5% vs. 60.2%, P=0.007) (74). Based on the data from midostaurin and sorafenib, the efficacy of quizartinib, gilteritinib, and crenolanib in maintenance therapy after HSCT will be evaluated in clinical trials (NCT02668653, NCT02997202, NCT02400255).

#### 3.1.3 The relapsed/refractory setting

Response rates are low in adult patients with relapsed/refractory (R/R) AML, and no standard strategy has emerged for treating primary refractory or relapsed AML (13). Patients with relapsed or refractory FLT3-mutated AML in the phase III ADMIRAL trial were randomly assigned to the subgroups of single-agent gilteritinib or salvage chemotherapy. Compared to salvage chemotherapy, gilteritinib monotherapy resulted in a higher percentage of patients with complete remission and full or partial hematologic recovery (34% vs. 15.3%) and improved OS with or without censoring at HSCT (9.3 months vs. 5.3 months, P<0.001) (23). The promising results of the established trial prompted FDA approval of gilteritinib in the R/R setting. Similarly, in the phase III QUANTUM-R trial, patients in the subgroup of single-agent quizartinib had an improved OS of 6.2 months versus 4.7 months (P=0.02) and a hazard ratio for death of 0.76 (95% CI: 0.58–0.98). However, complete remission rate did not improve from single-agent quizartinib (23). A *post hoc* analysis of the ADMIRAL and QUANTUM-R trials demonstrated that quizartinib treatment achieved remission faster and response may be more durable and survival potentially longer with gilteritinib regardless of substantial limitations in cross-study comparisons (75). Furthermore, the combination of venetoclax with gilteritinib showed a potential for molecular clearance, which seemed to be associated with increased OS in a recent trial (76). The promising latent capacity of FLT3 inhibitors in the re-lapsed/refractory setting has been demonstrated, and further exploration is ongoing (NCT03989713, NCT04140487, NCT05010122).

### 3.2 Therapy for non-canonical FLT3 mutated AML

With the clinical application of NGS methods over recent years, an increased number of FLT3 mutations outside of the ITD and D835/I836 regions have been de-scribed (16, 18, 77, 78). These non-canonical FLT3 mutations, including point mutations and small insertions/deletions, occur in every FLT3 protein domain and frequently in the juxtamembrane (JM) domain and the KD domain adjacent to D835/I836. Several studies have validated that many of the mutations acquire increased phosphorylation activity, and some of them might confer resistance or high sensitivity to specific FLT3 inhibitors (16, 29, 78, 79).

A series of point mutations located in the non-TKD region tend to result in aberrant phosphorylation of FLT3 and an enhanced ability to activate STAT5. Several point mutations in the TKDs were less sensitive to the type II inhibitor quizartinib than to the type I TKI crenolanib (80). In contrast, small insertions/deletions of FLT3, such as p.Glu598_Tyr599del and p. Phe590_Asp593delinsLeuTyr did not show an increase in Y589/Y591 phosphorylation but led to constitutive activation of STAT3, ERK1/2, SFK (Src family kinases), SHP2 and AKT. Interestingly, p.Phe590_Asp593delinsLeuTyr-transduced cells showed a higher sensitivity toward PKC412 and AC220 than FLT3-ITD-transduced cells (17).

The clinical characteristics of non-canonical mutations remain unclear because of their low incidence. In a brief report, a patient with mutation of p. Glu598_Tyr599del, which is described in the above study, is likely to be responsive to targeted therapy with tyrosine kinase inhibitors (77). Another report demonstrated that patients har-boring activating non-canonical FLT3 mutations (V592G and N676K) may benefit from TKI therapy in the relapsed/refractory setting (81). However, a recent study revealed that FLT3 non-canonical mutations in the ITD region have a higher rate of concomitant mutation of KTM2A-PTD, and the patients with dual FLT3 non-ITD and KMT2A-PTD mutations may indicate a trend for inferior outcome (82). The phosphorylation activity and clinical characteristics of non-ITD and TKD FLT3 mutations vary widely, but most of them may be sensitive to FLT3 TKIs.

## 4 Resistance to FLT3 inhibitors

**Figure 2:**
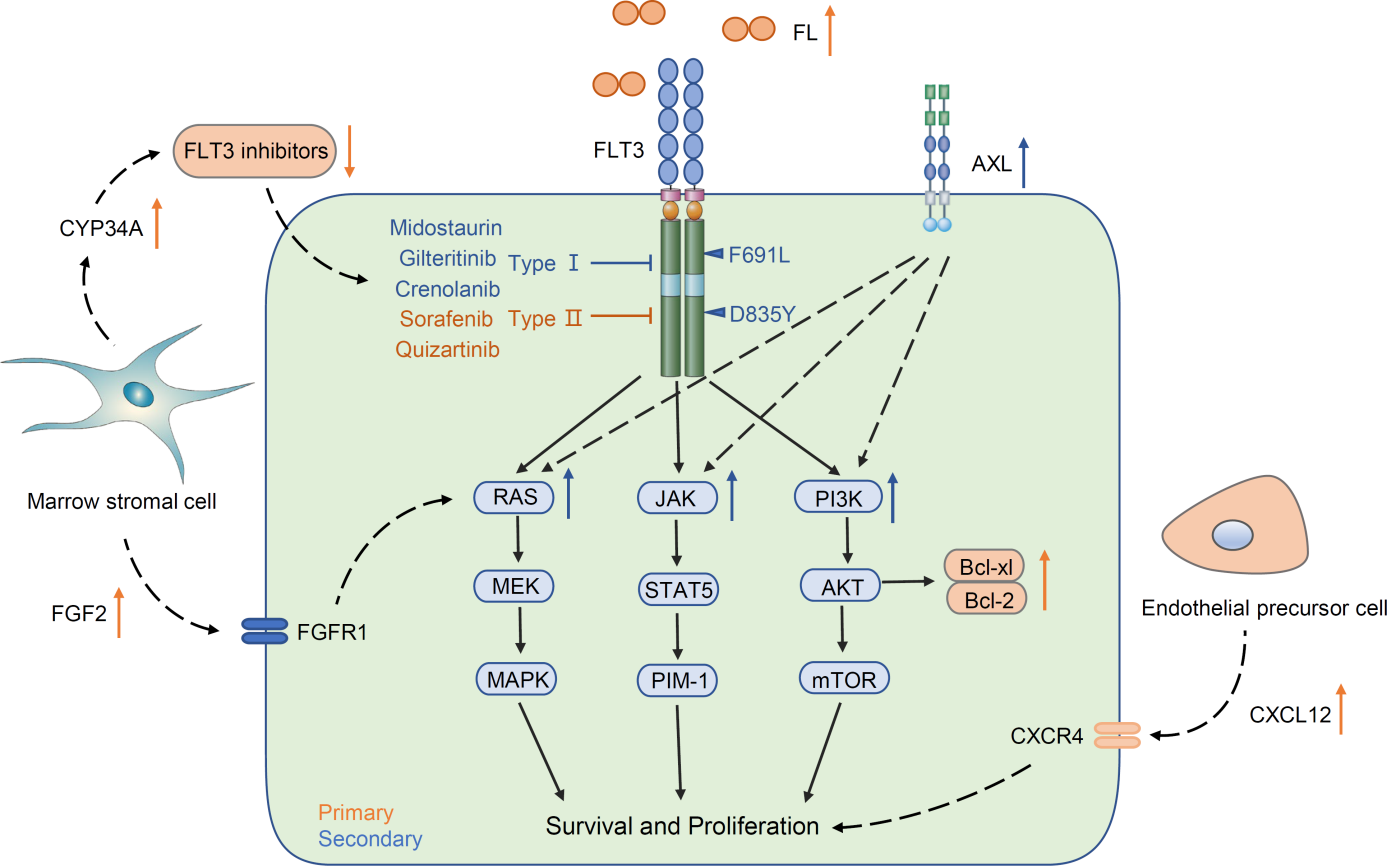
Common mechanisms of primary and secondary resistance.

Although multiple small molecule inhibitors of FLT3 have rapidly improved the outcomes of FLT3-mutated AML, resistance to FTL3 inhibitors has become increasingly prominent. Frequent short-lived responses and therapeutic resistance pose an ongoing issue. Resistance mechanisms, including primary resistance and secondary resistance, vary due to drug type. Primary resistance is considered innate. For example, overexpression of FL and the abnormal status of the marrow microenvironment induce resistance when FLT3 inhibitors are used for the first time. Secondary resistance defines the resistance that occurs after using FLT3 inhibitors, including the second mutation of FLT3 (on-target) and a non-FLT3 abnormality (off-target), such as acquiring an alteration of downstream and parallel signal pathways (**Figure 2**).

### 4.1 Primary resistance

One common mechanism of primary resistance is the increased FLT3 ligand. FLT3 ligand is an important regulator in hematopoiesis. FL is expressed by most leukemic cells and promotes proliferation through an autocrine process (83, 84). The soluble FL concentrations increase with the courses of chemotherapy and maintain a high level and increase more rapidly in relapse patients. Furthermore, increased plasma FL levels are tightly correlated with decreased efficacy of FLT3 inhibitors *in vitro* and *in vivo* (85). It is connected to increased FL that overexpression of FLT3 in AML blast cells can clearly impair the efficacy of FLT3 inhibitors. In addition, Bcl-2 is located down-stream of the FLT3/PI3K pathway and plays a significant anti-apoptotic role. The ab-normal elevation of Bcl-2 in FLT3-ITD-positive blasts protects tumor cells from apoptosis, and the Bcl-2 protein level is not decreased when FLT3 inhibitors attenuate the phosphorylation of FLT3 (86–88). Similar to Bcl-2, activation of other anti-apoptotic proteins, such as Mcl-1 and Bcl-xl, mediates resistance (36, 88).

The bone marrow microenvironment can also directly contribute to primary FLT3 inhibitor resistance. The upregulation of CXCL12 and fibroblast growth factor 2 (FGF2), which are secreted by endothelial precursor cells and bone marrow stromal cells in the bone marrow, respectively, can shield AML blasts from FLT3 inhibitor injury (89, 90). Hepatic CYP3A4 has also been shown to inactivate all TKIs and provide chemoprotection. Enhanced CYP34A expression on BM stromal cells will weaken the drug availability of FLT3-TKIs, leading to limited efficacy (91).

### 4.2 Secondary resistance

#### 4.2.1 On-target resistance

Secondary resistance can be broadly subdivided into on-target and off-target mechanisms according to where the alteration occurs. The most common on-target resistance is the development of the second FLT3 mutation, often in the KD (**Table 1**). Acquisition of point mutations at four residues (F691, D835, Y842, E608) in the kinase domain of FLT3-ITD confers resistance to quizartinib by disrupting binding (92). The gatekeeper F691L mutation and D835 mutation confer resistance not only to quizartinib but also to sorafenib, gilteritinib, and crenolanib (93). After using midostaurin, the TKD1 mutations N676D/S, F691I/L, and G697R/S were screened for efficacy (34). In these acquiring point mutations, D835 alterations tend to confer drug tolerance to type II FLT3 inhibitors by disrupting the binding and keeping the A-loop in a DFG-out state (94, 95). In contrast, the acquisition of the gatekeeper F691L and G697R/S mutations often means tolerance to most FLT3 inhibitors (34).

#### 4.2.2 Off-target resistance

On-target mutations only partly explain FLT3 inhibitor resistance, and upregulation or emergence of non-FLT3 mutant clones (off-target) is a key resistance mechanism (**Table 1**). Recent studies have demonstrated obvious differentiation in paired patients be-tween previous and posttreatment gilteritinib and crenolanib resistance. Upregulation of the Ras/MAPK pathway occurs frequently, causing resistance generation (48, 52). Using patient-derived cell lines, Lindblad O et al. and Knapper S et al. revealed an enrichment of the PI3K/mTOR and JAK/STAT5 signaling pathways in resistant cells (96, 97). The above evidence clearly shows that the aberrant activation of down-stream signaling pathways of FLT3 enables AML cells to become tolerant to FLT3 inhibitors.

Abnormal upregulation of parallel AXL tyrosine kinase signaling is another mechanism of FLT3 inhibitor resistance. AXL is a member of the TAM family with the high-affinity ligand growth arrest-specific protein 6 (GAS6). Activation of the GAS6/AXL signaling axis serves as an important pathway driving cancer cell survival and proliferation, which is similar and parallel to the FLT3 signaling pathway (98). In one study, AML cell lines and primary blasts from FLT3-ITD-mutated AML patients were treated with PKC412 and AC220 concomitantly, and the expression of phospho-AXL and AXL was detected. Enhanced phosphorylation of AXL by treatment with PKC412 and AC220 occurred not only in AML cell lines but also in primary blasts (37). Similarly, the results from another study validated that the increased level of AXL dampened the response to the FLT3 inhibitor quizartinib. In a xenograft mouse model of this study, inhibition of AXL significantly enhanced the response of FLT3-ITD cells to quizartinib exclusively within a bone marrow environment (51). These data high-light a new bypass mechanism that attenuated the response to FLT3 inhibitors through upregulation of AXL activity.

#### 4.2.3 Other mechanisms of secondary resistance

**Figure 3:**
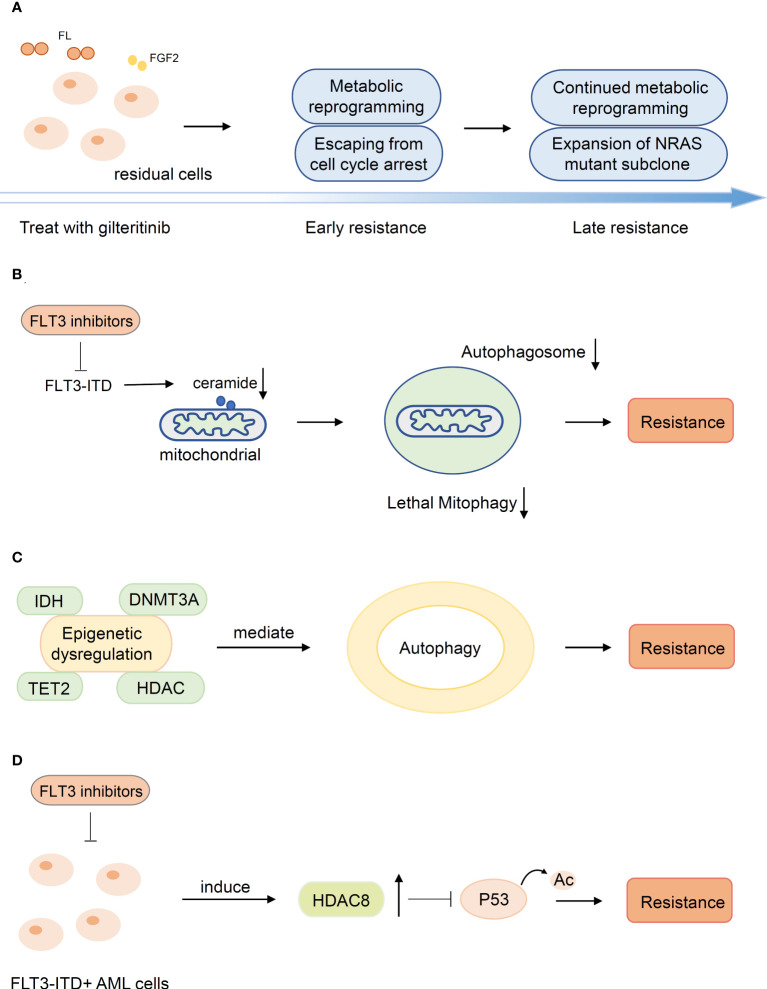
Other mechanisms of secondary resistance. **(A)** Metabolic reprogramming mediated the evolution of resistance. **(B)** The abnormality of mitochondrial ceramide results in resistance to FLT3-inhibitors by arresting mitophagy. **(C)** Mutations in epigenetic modification genes suggests potential associations between alterations and autophagy associated with secondary resistance **(D)** HDAC8 was upregulated upon FLT3 inhibitor exposure and enhanced HDAC8 binding and deacetylation of p53 to promote resistance.

The emergence of metabonomics has prompted the exploration of the mechanisms driving FLT3-ITD acute myeloid leukemia resistance to FLT3 inhibitors from a new perspective. Metabolic reprogramming mediated the evolution of gilteritinib resistance (**Figure 3A**). Sunil K Joshi et al. demonstrated dramatic differences in the metabolome of early and late gilteritinib-resistant AML cell lines. Metabolic profiling of these cells affirmed a trend toward upregulation of sphingolipid/phospholipid or fatty acid/carnitine metabolites relative to MOLM14 parental cells. Early resistant cells undergo metabolic reprogramming with a slow proliferation rate, while expansion of pre-existing NRAS mutant subclones is dominant in late resistant cells (99). The role of autophagy in targeted therapy has gradually been revealed. Enhanced autophagy activity was observed in sorafenib-resistant AML cell lines bearing FLT3-TKD mutations and FLT3-ITD cells participating in AML progression and drug resistance (100, 101). Although the molecular mechanisms remain a mystery, it is highly probable that the transcription factor ATF4 (activating transcription factor 4) stimulated by FLT3-ITD is crucial to the upregulation of autophagy (102). Mitophagy is a cellular process for the degradation of mitochondria by the autophagic machinery and is regulated by ceramide on the outer mitochondrial membrane (103). FLT3-ITD mutations rescue AML cells from mitophagy by suppressing pro-cell death lipid ceramide generation, and FLT3-ITD inhibition mediates ceramide-dependent mitophagy, leading to AML blast death. The abnormality of mitochondrial ceramide arresting mitophagy results in resistance to FLT3-ITD inhibition (104) (**Figure 3B**).

Epigenetic dysregulation is a significant cause of secondary resistance. Mutations in epigenetic modification genes (DNMT3A, TET2, IDH1/2, HDAC) are related to diverse processes of epigenetic regulation and suggest potential associations between alterations and autophagy associated with secondary resistance (48, 102, 105) (**Figure 3C**). In addition, HDAC8 was upregulated upon FLT3 inhibitor exposure in FLT3-mutated AML cells and subsequently enhanced HDAC8 binding and deacetylation of p53 to promote AML cell survival and TKI resistance (106, 107) (**Figure 3D**).

## 5 Strategies to overcome resistance

Numerous efforts have been made to attenuate the effect of FLT3 TKI drug tolerance. To overcome primary and off-target resistance, doctors attempt to combine different types of agents and use multitarget inhibitors to avert limited response. Due to on-target resistance, it is necessary to develop new FLT3 inhibitors that are effective against primary and secondary mutations *via* an original molecular design rationale.

### 5.1 Development of novel FLT3 inhibitors

As described above, type II inhibitors exhibit high selectivity but no response to FLT3-TKD mutation because of a persistent state of DFG-out (95). Although type I inhibitors show efficacy in overcoming FLT3-TKD, several secondary FLT3 mutations, for example, the gatekeeper mutation (F691L), confer limited efficacy to all existing FLT3 inhibitors (95). To tackle this challenge, the discovery of novel agents that are sensitive to secondary mutations conferring resistance is essential. The development of new type II inhibitors seemed to bring promising prospects. Pexidartinib (PLX3397) is a triple-kinase inhibitor of FLT3, KIT, and CSF1R and shows excellent inhabitation of FLT3-ITD (half-maximal inhibitory concentration [IC50]: 11 nM). Pexidartinib has a shorter linker between the middle and tail pyridine rings than other type II inhibitors and forms two hydrogen bonds with D829, which sequesters pexidartinib away from F691, thus avoiding the adverse influence of the F691L mutation. Extraordinary efficacy in overcoming the secondary F691L mutation has been shown due to the above mechanism (108, 109). Ge et al. discovered a novel type II FLT3 inhibitor, MZH29, which can stably bind to the FLT3-F691L model by four hydrogen bonds formed between MZH29 and E661, C694, and D829 and a π−π stacking interaction formed between MZH29 and F830 (110). Another novel FLT3 inhibitor, cabozantinib, potently inhibited FLT3 phosphorylation in FLT3-ITD-positive and FLT3-ITD-F691L-positive cells under both medium and plasma conditions, and the result of the docking binding model in which cabozantinib maintained a remote distance from F691 and formed a hydrogen bond with D829 opposite F691 explains the potent inhibitory activity (111). All these novel type II inhibitors, which noncovalently bind to the FLT3-ITD-F691L model, show stable binding ability and excellent efficiency *in vitro*. However, they were vulnerable to other residue mutations, particularly the residue located in the activation loop (108, 110, 111). Consequently, targeted covalent inhibitors have attracted the attention of many researchers. FF-10101, the first reported covalent FLT3 inhibitor, made great progress in enhancing the binding affinity and response to secondary on-target mutations by targeting conserved amino acids. A covalent bond was generated between the warhead of FF-10101 and the “SH” of C695.70 Based on its high affinity for FLT3 kinase, FF-10101 showed outstanding efficacy against not only the FLT3-ITD-TKD mutation but also the uncommon K663Q, N841I, R834Q, and F691L mutations *in vitro*. Furthermore, oral administration in NOD/SCID mice also showed potent inhibitory effects (112). FF-10101 is now in a phase I/II clinical trial (NCT03194685) to treat refractory or relapsed AML patients in the US.

### 5.2 Therapies of multitarget inhibition

Monotherapy with FLT3 inhibitors frequently causes drug resistance, a short duration of remission, and poor effects due to complex pathological changes, such as an abnormal microenvironment using FLT3 inhibitors and compensatory off-target alterations. That many non-FLT3 abnormalities are involved in the occurrence of FLT3 inhibitor tolerance indicates that simultaneously inhibiting multiple synergetic signaling molecules might contribute to improving the efficacy and overcoming resistance. Much evidence has demonstrated that the combination of different kinds of agents according to various abnormal states results in promising outcomes.

The combination of midostaurin or gilteritinib with the Bcl-2 inhibitor venetoclax contributed to the simultaneous downregulation of Mcl-1 and Bcl-2, resulting in the synergistic induction of apoptosis and attenuating the adverse impact of increased Bcl-2 (113, 114). Similarly, the concurrent inhibition of CXCL12 and FGF2 could have a favorable effect (89, 90). Furthermore, a synergistic effect has been shown in therapies combining FLT3-TKIs with agents targeting downstream or parallel signaling pathways, such as JAK/STAT5 pathway inhibitors (115, 116) and PI3K/mTOR pathway inhibitors (99, 117). In addition to combination therapies, dual inhibitors can achieve good results by reaching the identical goal of multitarget inhabitation, such as dual Pim kinase/FLT3 inhibitors, dual FLT3/JAK2 inhibitors, and dual FLT3/CDK4 inhibitors (118–122).

Moreover, addressing metabolic abnormalities is also a potential strategy to drive AML cells to return to a susceptible state (99, 104).

## 6 Conclusions and future directions

In the era of individual diagnosis and treatment, FLT3-activating mutations are a marker of poor prognosis and specific therapeutic needs. Although the emergence of FLT3 inhibitors has provided us with numerous powerful creative treatment tools, survival remains poor in FLT3-mutated AML, and new strategies need to be explored. In addition to FLT3-ITD/TKD, FLT3 non-canonical alterations also deserve attention with the wide use of NGS. At the same time, the influence of FLT3 inhibitor tolerance has become an inevitable issue. To solve this problem, a deeper exploration of the underlying mechanisms and resolvents from many different angles are essential.

## Author contributions

S-SG wrote the manuscript. S-BL and S-LX conducted review and editing. All authors have read and agreed to the published version of the manuscript.

## Funding

This work was supported by the grants from the National Natural Science Foundation of China (grant No. 81970138), Translational Research Grant of NCRCH (grant No. 2020ZKMB05), Jiangsu Province “333” project, Social Development Project of the Science and Technology Department of Jiangsu (Grant No. BE2021649) and Gusu Key Medical Talent Program (grant No. GSWS2019007). Key technology program of Suzhou people's livelihood technology projects (Grant No. SKY2021029).

## Conflict of interest

The authors declare that the research was conducted in the absence of any commercial or financial relationships that could be construed as a potential conflict of interest.

## Publisher’s note

All claims expressed in this article are solely those of the authors and do not necessarily represent those of their affiliated organizations, or those of the publisher, the editors and the reviewers. Any product that may be evaluated in this article, or claim that may be made by its manufacturer, is not guaranteed or endorsed by the publisher.
